# A high-throughput drug combination screen of targeted small molecule inhibitors in cancer cell lines

**DOI:** 10.1038/s41597-019-0255-7

**Published:** 2019-10-29

**Authors:** Åsmund Flobak, Barbara Niederdorfer, Vu To Nakstad, Liv Thommesen, Geir Klinkenberg, Astrid Lægreid

**Affiliations:** 1 Department of Clinical and Molecular Medicine, Norwegian University of Science and Technology, Trondheim, Norway; 2 The Cancer Clinic, St. Olav’s Hospital, Trondheim, Norway; 3 SINTEF Materials and Chemistry, Department of Biotechnology, Trondheim, Norway; 4 Department of Biomedical Laboratory Science, Norwegian University of Science and Technology, Trondheim, Norway

**Keywords:** High-throughput screening, Targeted therapies

## Abstract

While there is a high interest in drug combinations in cancer therapy, openly accessible datasets for drug combination responses are sparse. Here we present a dataset comprising 171 pairwise combinations of 19 individual drugs targeting signal transduction mechanisms across eight cancer cell lines, where the effect of each drug and drug combination is reported as cell viability assessed by metabolic activity. Drugs are chosen by their capacity to specifically interfere with well-known signal transduction mechanisms. Signalling processes targeted by the drugs include PI3K/AKT, NFkB, JAK/STAT, CTNNB1/TCF, and MAPK pathways. Drug combinations are classified as synergistic based on the Bliss independence synergy metrics. The data identifies combinations that synergistically reduce cancer cell viability and that can be of interest for further pre-clinical investigations.

## Abstract (summary)

**Table:** 

Measurement(s)	regulation of response to drug • cancer cell line • ATP quantitation using luciferase • cell density determination
Technology Type(s)	high throughput sample analysis • CellTiter-Glo Luminescent Cell Viability Assay • imaging cytometer
Factor Type(s)	cell line • biological replicate • (5Z)-7-oxozeaenol • doramapimod • CHIR 99021 • D4476 • GSK2334470 • GSK-429286 • PD0325901 • PI-103 • toxoflavin • ruxolitinib • stattic • drug
Sample Characteristic - Organism	Homo sapiens

Machine-accessible metadata file describing the reported data: 10.6084/m9.figshare.9963797

## Background & Summary

Treatment of non-resectable cancer has previously mainly relied on cytotoxic chemotherapy that indiscriminately kills all rapidly dividing cells. With the discovery of molecular mechanisms driving cancer, a new generation of molecularly targeted drugs has entered the market over the last two decades. A remaining challenge in the envisaged transition from ‘one-size-fits all’ therapeutic approach to personalised treatment is the development of resistance to single-targeted drug treatment. Combinatorial therapy may be able to overcome this by co-targeting multiple mechanisms involved in cancer cell growth and survival^1,2^.

Already compelling results have been achieved with some combinations of cancer signalling inhibitors, e.g. the synergistic combination of MAP2K1/2 (MEK) and BRAF inhibitors for malignant melanoma^3–5^ and lung adenocarcinoma^6^. This has sparked a wide interest in identifying novel efficient pairs of small molecule targeted drugs. However, our knowledge about beneficial targeted drug combinations is still limited, partly due to the combinatorial complexity.

Few high-throughput screens testing multiple targeted drugs in combinations have been published with open access data. Examples include combinatorial screens on ovarian cancer cell lines^7^, melanoma cell lines^8^, sarcoma cell lines^9^ and lung-cancer-patient-derived cell culture models^10^. In a study performed by O’Neil *et al*., combinations of around 12 targeted drugs were screened across a panel of 39 cancer cell lines. In total 22 experimental drugs were tested in all possible combinations and in combination with 16 approved drugs^11^. The National Cancer Institute (NCI) screened pairs of 104 FDA-approved cancer drugs against the NCI-60 cell line panel. Around 30 of the tested compounds can be classified as small molecule targeted therapies^12^. In the AstraZeneca’s drug combination data set of the latest DREAM challenge, 118 drugs, including 59 targeted therapies, were tested in 910 pairwise combinations against 85 cancer cell lines^13^.

**Fig. 1:**
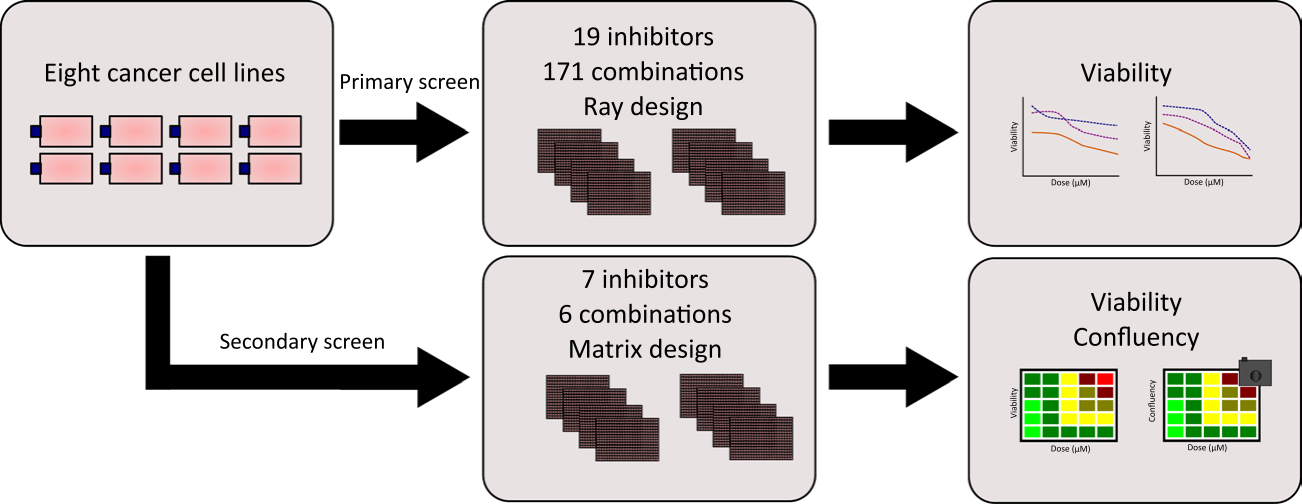
Schematic representation of study design. Eight human cancer cell lines from different tissue origins were used in this study. Cells were incubated overnight prior to drug addition. In the primary screen, cells were screened against 19 small-molecule inhibitors in single and double application, with a total of 171 combinations. After 48 hours of drug exposure the assay was terminated, and cell viability was measured using CellTiter-Glo 2.0 (Promega). In the secondary screen, cells were screened against 7 single small-molecule inhibitors and 6 combinations for a duration of 48 hours. Drug effect was measured using automated brightfield imaging of confluency and CellTiter-Glo 2.0 (Promega).

In this paper, we report results from a high-throughput screen testing the effects of combining 19 small-molecule inhibitors on cancer cell viability. The drugs target signalling proteins involved in several well described signalling pathways, including PI3K/AKT, NFkB, JAK/STAT, CTNNB1/TCF, and MAPK pathways. Inhibitors were selected for high specificity and a minimum of known off-target activities, as judged from characterizations available from the MRC PPU lab (https://www.ppu.mrc.ac.uk/)^14^ for 15 of 19 drugs. Eight cancer cell lines were treated with single inhibitors and 171 pairwise combinations at equimolar concentrations from 10–0.16 µM. Cell viability was determined after 48 hours from ATP content using CellTiter-Glo 2.0 (Promega). Several of the synergistic combinations involved the PI-103 inhibitor. Six of these combinations were investigated in a secondary screen. We here applied a matrix design allowing for synergy assessment outside the equimolar range and determined inhibitor effects from both viability (ATP content) and cell confluency (brightfield imaging). Figure 1 gives a schematic overview of the study design.

In the here presented dataset we rediscovered previously reported synergistic combinations and discovered several new synergies. The synergistic reduction of cell viability resulting from jointly targeting PI3K/mTOR and TAK1, previously reported by us for the AGS cell line^15^ and later by others^16^, is observed in two of the tested cell lines. Furthermore, we rediscovered the synergistic effect of jointly targeting MEK and PI3K^17–19^. Recently, Sathe *et al*. have reported on increased effect of combined inhibition of PI3K/mTOR and PDPK1 in bladder carcinoma cells^20^. We identify that combined targeting of PI3K/mTOR and PDPK1 is potent across multiple cancer cell lines. The PI3 kinase is one of the canonical activators of PDPK1, and our dataset thus identifies this ‘vertical synergy’ intervention by targeting multiple points in one cascade as a potential chemical intervention for inhibiting PI3K-dependent cancer growth across cell line models representing different tissue types. Co-targeting of multiple vertical points in one cascade is also the rationale for the clinically verified ‘vertical synergy’ observed by jointly targeting MEK and BRAF^3–6^. Moreover, PI3K and PI3K/mTOR inhibitors tested in a clinical setting (e.g. pictilisib^21^ and apitolisib^22^) have suffered from toxic effects^23,24^. Thus, identification of compounds that increase the therapeutic index for PI3K inhibitors could enable new uses for PI3K inhibition in oncology.

The outcomes of our work contribute to expanding the available data sets on novel combinations of targeted drugs across a variety of cancer cell lines and can benefit the fields of cancer therapeutics, molecular signalling interventions and molecular cancer biology.

## Methods

### Cell lines and culturing

The following eight cancer cell lines were used in this study: A498 (kidney cancer), AGS (gastric adenocarcinoma), COLO 205 (colorectal cancer), DU-145 (prostate cancer), MDA-MB-468 (breast cancer), SF-295 (glioblastoma), SW-620 (colorectal cancer), and UACC-62 (melanoma). All cell lines used in the study are of human origin. Seven of the cell lines were obtained from the NCI-DCTD Repository, Frederick MD, and are part of the DTP 60 cell line panel (NCI-60 collection). The AGS cell line was obtained from ATCC. AGS cells were cultured in HAMS’s F12 (GIBCO, 21765-029) supplemented with 10% FBS (GIBCO, 10270-106), 10 µg/ml penicillin-streptomycin (GIBCO, 15140-122) and 1 µg/ml fungizone (GIBCO, A11138-03). MDA-MB-468 were cultured in RPMI 1640 (GIBCO, 31870-025) supplemented with 5% FBS (SIGMA, F7524), 2 mM L-Glutamine (SIGMA, G7513) and 100 U/ml penicillin-streptomycin (GIBCO, 15140-122). All other cells were cultured in RPMI 1640 (GIBCO, 31870-025) supplemented with 10% FBS (SIGMA, F7524), 2 mM L-Glutamine (SIGMA, G7513) and 100 U/ml penicillin-streptomycin (GIBCO, 15140-122). Cells were cultured for approximately two weeks before assay and discarded after a maximum of two months in culture. The day before seeding out cells for the high throughput assay, cells were sub-cultured 1:2. Due to technical reasons, this was not performed for biological replicate 2 and 3 of the follow-up secondary screen.

### Primary screen - ray designed drug treatment

Cell lines were treated for 48 hours with 19 small-molecule inhibitors in single and pairwise applications, comprising a total of 171 drug combinations. All drugs were pre-diluted in DMSO to a stock concentration of 20 mM. Drugs were applied in an equimolar ray design from 10 µM to 0.016 µM using the following procedure: First, 2.0 mM solutions of each inhibitor and inhibitor combination were prepared. These were then serially diluted in DMSO (four 5-fold serial dilutions). The solutions were further diluted in medium (8 µl inhibitor in DMSO in a total volume of 228 µl), of which 5 µl were added to each cell culture well. Total cell culture volume per well was 35 µl. Highest final concentrations were thus 10 µM in each well. All dilutions and liquid handling were performed with robotic equipment using sterile and pyrogen free pipette tips. The inhibitors are listed in Online-only Table 1. Final DMSO concentration in assay wells was 0.5%.

### Secondary screen - matrix designed drug treatment

In the secondary screen six combinations were screened in a matrix designed across all cell lines. As the PI inhibitor was identified as one of the top drugs involved in synergistic combinations, it was chosen as the anchor drug. The inhibitor was combined with BI, D1, JN, G2, PD, and 5Z. Cell lines were treated for 48 hours with the seven small-molecule inhibitors in single application and six drug combinations in all possible combinations of doses 10, 2, 0.4, 0.08 and 0.02 µM. Drugs, prepared as stocks of 20 mM in DMSO, were first diluted to 400x final concentration in DMSO. The solutions were further manually mixed 1:1 with either DMSO, for single drugs, or combination drug and applied to a 96 well plate. Further liquid handling was performed with robotic equipment. The solutions were diluted in medium (28.5x), of which 5 µl was added to each cell culture well leading to a total volume of 35 µl. We note that a new set of inhibitors was ordered for this screen.

### High-throughput screen

Cells were seeded in 384-well plates (CORNING, 3712) at densities shown in Table 1 and allowed to attach overnight, before they were treated with small-molecule inhibitors and incubated for another 48 hours. The cell seeding densities were selected such that the cells were in a sub confluent stage at the time of drug addition. The variances in doubling time between the cell lines were taken into account and seeding densities were adjusted so that cell cultures in wells were either in late log phase or early plateau at the end of the incubation period. Cell viability was measured using the commercially available CellTiter-Glo 2.0 assay (Promega). In the secondary screen confluency was included as an additional readout and monitored by brightfield imaging using (SpectraMax i3x MiniMax 300 Imaging Cytometer, 2 views per well). A set of control wells with cells treated with 0.5% DMSO was included on all plates. The assay was performed with three biological replicates, if not stated otherwise.

**Table 1:** Cell lines used in screen.

Cell line	Tissue of origin	Cells/well (384-well plate)	Population doubling time (h)	Growth Media during drug screening	Term Accession Number	Term Source REF
A498	kidney cancer	972	66.8	RPMI 1640 10%FBS	BTO:0003769	BTO
AGS	gastric adenocarcinoma	800	20	HAMS’s F12 5%FBS	BTO:0001007	BTO
COLO 205	colorectal cancer	3000	23.8	RPMI 1640 10%FBS	BTO:0000179	BTO
DU-145	prostate cancer	2100	32.3	RPMI 1640 10%FBS	BTO:0001332	BTO
MDA-MB-468	breast cancer	8100	62	RPMI 1640 5%FBS	BTO:0001570	BTO
SF-295	glioblastoma	1050	29.5	RPMI 1640 10%FBS	BTO:0004213	BTO
SW-620	colorectal cancer	3750	20.4	RPMI 1640 10%FBS	BTO:0000675	BTO
UACC-62	melanoma	945	31.3	RPMI 1640 10%FBS	BTO:0004152	BTO

Cell line density and growth media used are indicated. All media were supplemented with L-glutamine and penicillin-streptomycin. Population doubling time as annotated by provider. Cell lines are identified by BRENDA tissue/enzyme source (BTO, Version: 2016-05-05).

For the first two biological replicates of the primary screen, two technical replicates are reported. Two of the four technical replicates assayed were excluded due to insufficient mixing of drug dilutions for these replicates. For the third biological replicate all four technical replicates assayed are included. For MDA-MB-468 and AGS cells, one or two biological replicates were performed, respectively. Some data points for the AGS cell line had to be excluded due to erroneous drug addition within the robotic script.

For the first biological replicate in the secondary screen, some technical replicates had to be excluded for the SW-620 cell line as too little drug volume was added. For the MDA-MB-468 cell line the first biological replicate was excluded due to low cell viability in the control wells. For the first, second and third biological replicate some technical replicates had to be excluded due to non-efficient cell attachment caused by an air bubble below the cell suspension. Additionally, some wells were excluded in the cell confluency data due to condensation of water on the bottom of the plate.

### Data processing and representation

#### Viability data - Primary screen

**Fig. 2:**
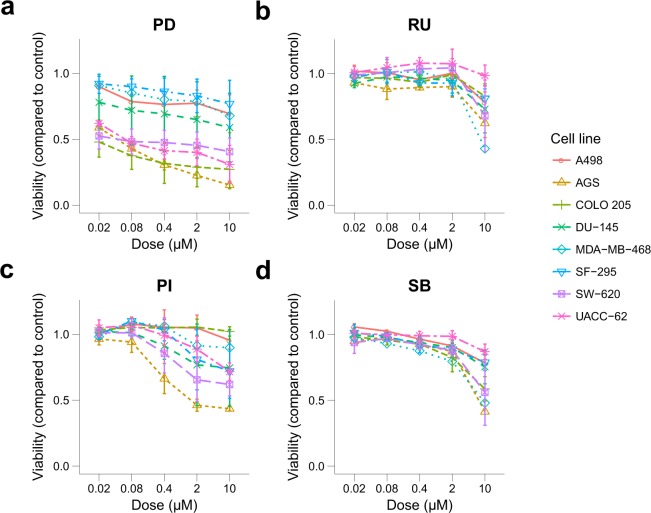
Single dose response data of 4 out of 19 tested drugs across eight cell lines. The plot shows the viability of the eight tested cancer cell lines exposed to four different inhibitors in single application in a 5-fold dilution series from 10–0.016 µM, with standard deviation indicated by error bars. Viability was normalised to DMSO control. Abbreviations: PD-0325901 (PD); Ruxolitinib (RU); PI-103 (PI); SB-505125 (SB).

For each 384-well plate, viability data of treated cells was normalised to the DMSO control cells measured in the same plate. Normalised viability data for each biological replicate where processed using “R-script-average-of-biological-replicates.R”. The average viability score for each technical replicate as well as the average viability score across the biological replicates were calculated and exported as tab-separated values (tsv) files. Standard deviation across biological replicates was calculated using “R-script-calculate-mean-STDV.R”. Dose response curves were generated using “R-script-sensitivity-of-drugs-across-cell-lines-plot.R” (Fig. 2, and in figshare deposit^25^: Drug_cell_response.pdf). Drug combination plots were generated from average viability scores with standard deviations using “R-script-plotting-drug-combination.R” (figshare deposit^25^: Combination_plots_primary_screen.pdf). Data points with missing values (14%) are represented as *NA* values.

#### Viability and confluency data - Secondary screen

For confluency data brightfield images were analysed using the SoftMax® Pro Software field analysis setting. For this, the region of the image covering the well was selected and confluency was reported. We note that the absolute values for confluency for very flat and long cells (e.g. A498, SF-295) may be underestimated while absolute confluency for round cells (e.g. MDA-MB-498) may be overestimated. We further want to indicate that COLO 205 are growing sub-confluent. For completeness of the data set, confluency is reported for this cell line. For each 384-well plate, viability and confluency data of treated cells was normalised to the plate-internal DMSO control. Technical replicates and averages of biological replicates are reported as comma-separated files (csv). These were further processed using “Biological_Average_secondary_screen.R” to calculate the biological average, standard deviation and coefficient of variation. Drug combination plots were generation from average viability scores with standard deviations using “Combination_plots_secondary_screen.R” (figshare deposit^25^: Combination_plots_secondary_screen_viability.pdf). The same script was used to generate drug combination plots for confluency data (figshare deposit^25^: Combination_plots_secondary_screen_confluency.pdf).

#### Calculation of synergy and statistical significance of quantified synergy

Bliss excess was evaluated for all doses and biological replicates per drug combination and cell line. We first computed all possible Bliss expectations of the reported technical replicates per dose per drug combination. Each technical replicate of combination dose responses was then compared to the average of computed Bliss expectations per technical replicate per dose in same biological replicate. For calculation of Bliss viability values were capped at one.

$$Bliss\,excess:\,viability(drug\,A+drug\,B)-viability\,(drug\,B)\ast viability\,(drug\,A)$$

**Fig. 3:**
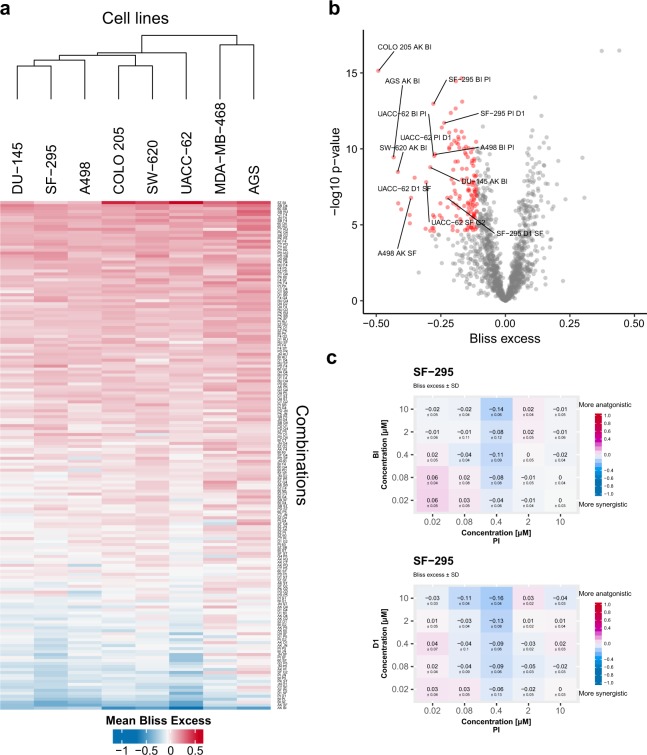
Overview of synergy scores of all drug combinations across all tested cell lines. (**a**) The heatmap shows Bliss excess across the eight cell lines tested in this study, where a negative value indicates a stronger synergy. Bliss excess ≤ −0.11 are coloured in blue (synergy), while values ≥ 0.11 are coloured in red (antagonism). Cell lines are clustered using Euclidian distances of synergy strengths, while rows are sorted according to mean Bliss excess across all cell lines. (**b**) Volcano plot showing synergy strength (Bliss excess) vs significance scores of synergistic responses. (**c**) Average Bliss excess ± standard deviation for SF-295 cells treated with Doramapimod (BI) in combination with PI-103 (PI) and BI-D1870 (D1) in combination with PI from secondary screen.

In the primary screen statistical significance of the synergistic response was evaluated. For this the computed Bliss excesses for each drug combination-cell line was compared to a zero-centred normal distribution. T-tests were performed to test for statistically significant deviations from a zero-centred distribution. When the null hypothesis was rejected the alternative hypothesis was accepted, which states that the distribution is not zero-centred, i.e. either antagonistic or synergistic. A conservative Bonferroni correction was done to adjust p values for multiple hypotheses. Combinations with average Bliss excess values ≤ −0.08 and p-value ≤ 0.05 were classified as synergies. Bliss excess and significance classifications were performed using “p_values_Bliss.R”. A synergy score heatmap and volcano plot visualising mean Bliss excess was generated using “R-script-heatmap.R” (Fig. 3a,b). For the secondary screen average Bliss excesses per dose, combination and cell line were computed using “Bliss_synergy_secondary_screen.R”. A cumulative Bliss score was calculated using the same script by taking the mean over the computed Bliss excesses per dose. Combinations with average Bliss excess value < 0 were considered as synergies. The lower cut-off was motivated by the fact that five times more combination concentrations were tested in the secondary compared to the primary screen. Example plots of mean Bliss excess ± standard deviation for two combination tested in the secondary screen are illustrated in Fig. 3c.

### Drug target annotation

Online-only Table 1 lists inhibitor abbreviation, name and unique identifiers (InChiKey and PubChem CID) along with the provider and primary targets as given by provider. CHEBI IDs are provided when available. We provide extensive target profile information in the form of bioassay information extracted from PubChem (last accessed on 24-06-2019) and kinase profiling data from the MRC PPU International Centre for Kinase Profiling - Kinase Profiling Inhibitor Database (http://www.kinase-screen.mrc.ac.uk/kinase-inhibitors, last time accessed 24-06-2019) available in our figshare deposit^25^.

For the inhibitors SF1670, BI605906 and 10058-F4 no bioassay information supporting targets given by the provider were found in PubChem or MRC PPU Inhibitor Database (proteins inhibited below 10% activity). For these inhibitors, we performed an exhaustive literature search to provide annotated information^25^.

## Data Records

All data is available at figshare^25^, 10.6084/m9.figshare.9810719. Below, the file names for the different data sets are listed.

### Viability and confluency data

**Fig. 4:**
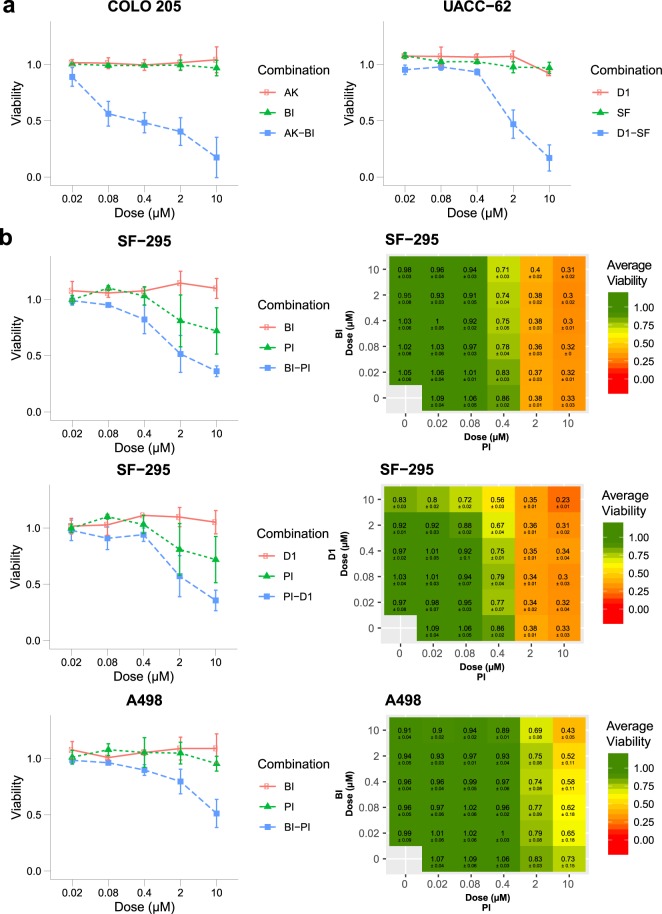
Example drug combination plots for drug combinations found to be highly synergistic according to Bliss excess and p-value. (**a**) The graphs show viability data with standard deviation for COLO 205 cells tested Doramapimod (BI) and Akt Inhibitor VIII (AK) and UACC-62 cells tested with BI-D1870 (D1) and SF1670 (SF) in the primary screen. (**b**) The graphs show viability data with standard deviation for SF-295 cells treated with drug combinations Doramapimod (BI) - PI-103 (PI) and BI-D1870 (D1) - PI, and A498 cells treated with BI - PI in the primary and secondary screen. The data is normalised to DMSO control.

The raw luminescence data from the three biological replicates from the primary screen can be found in NTNU_HTS_Repl[1-3]_raw.txt within figshare^25^. Relative viability data per biological replicate is available in [Cell line].NTNU_HTS_Repl[1-3]_processed.tsv^25^. Mean viability data of technical and biological replicates are depicted in [Cell line].tech-mean_Effect.tsv^25^ and [Cell line].mean_Effect.tsv^25^. Example plots for drug sensitivity across the eight cell lines are shown in Fig. 2, while all plots can be found in Drug_cell_response.pdf^25^. In Fig. 4, the combination effect of selected drug combinations is visualised. Visualised drug combinations are based on significance and strength of synergy (see section for *Calculation of synergy and statistical significance of quantified synergy*, Fig. 3). Figure 4b shows average viability of SF-295 cells and A498 cells treated with Doramapimod (BI) in combination with PI-103 (PI), and SF-295 cells treated with BI-D1870 (D1) in combination with PI from the primary and secondary screen. The complete file with all drug combination plots can be found in Combination_plots_primary_screen.pdf^25^ for the primary screen and in Combination_plots_secondary_screen_viability.pdf and Combination_plots_secondary_screen_confluency.pdf for the secondary screen for viability and confluency response, respectively^25^.

The raw luminescence data and relative viability data from the three biological replicates from the secondary screen can be found in NTNU_HTS_secondary_viability_REPL[1-3].csv^25^. Raw as well as relative confluency data can be found in NTNU_HTS_secondary_confluency_REPL[1-3].csv^25^. Mean viability and confluency across the three biological replicates are depicted in NTNU_HTS_secondary_viability_Biological_Average.tsv^25^ and NTNU_HTS_secondary_confluency_Biological_Average.tsv^25^, respectively.

### Synergy data

The Bliss excess values for each combination tested in the primary screen across the eight cell lines are represented in bliss_significance.tsv^25^. Synergistic drug combinations are further presented in bliss_synergies.tsv^25^. A visual representation of Bliss excess is depicted in Fig. 3a. Cell lines were clustered according to Euclidean distances of quantified synergy strengths. 4% synergies were recorded according Bliss Independence model.

Bliss excess values for each combination per dose tested in the secondary across eight cell lines are represented in bliss_synergies_secondary_screen_viability.tsv^25^ and bliss_synergies_secondary_screen_confluency.tsv^25^ from viability and confluency data, respectively. A visual representation of Bliss excess ± standard deviation from the viability data is shown in bliss_synergies_secondary_screen_plots_viability.pdf^25^ and in bliss_synergies_secondary_screen_plots_confluency.pdf^25^ from the confluency data. Cumulative bliss scores for the viability data can be found in Mean.Bliss.excess.matrix.tsv^25^.

## Technical Validation

### Cell line identity

The seven NCI-cell lines used were received from the NCI-DCTD Repository and cultivated no more than 25 passages after reception in the lab. All cell lines were validated by STR profiling performed by Cell Line Authentication Service at ATCC. Eight core STR loci (TH01, D5S818, D13S317, D7S820, D16S539, CSF1PO, vWA and TPOX) plus amelogenin were matched to database profiles obtained from ATCC and Deutsche Sammlung von Mikroorganismen und Zellkulturen GmbH (DSMZ). For two of the cell lines, SF-295 and UACC-62, no database profile was available for comparison from ATCC and DSMZ. However, both cell lines were found to match their STR profile listed on the ExPASy website (https://web.expasy.org/cellosaurus). All 7 cell lines were found to be identical or similar to their database profiles with percent matches at 95–100%. The AGS cell line was validated by STR profiling performed by the in-house Genomics Core Facility. Eight core STR loci (TH01, D5S818, D13S317, D7S820, D16S539, CSF1PO, vWA and TPOX) plus amelogenin had a match of 81% to the ATCC profile (CRL-1739).

### Viability assay performance and variation

The performance of the viability assays was monitored using a sample set of small molecule positive controls. In the ray design nine concentrations of digitonin ranging from 100 µg/ml to 0.02 µg/ml was included in each biological replicate. In the matrix design a sample set with three concentrations of the positive controls digitonin, ranging from 26 µg/ml to 3 µg/ml, and staurosporine, ranging from 1.6 µM to 0.18 µM, were included in each plate. The standard deviation of the plate internal reference groups was also monitored and was less than 10% at maximum (on average below 5%) in the ray designed screen. Overall, we observed a mean coefficient of variation of 8% across all biological replicates in the primary screen and 7% in the secondary screen for both the viability and confluency data.

**Fig. 5:**
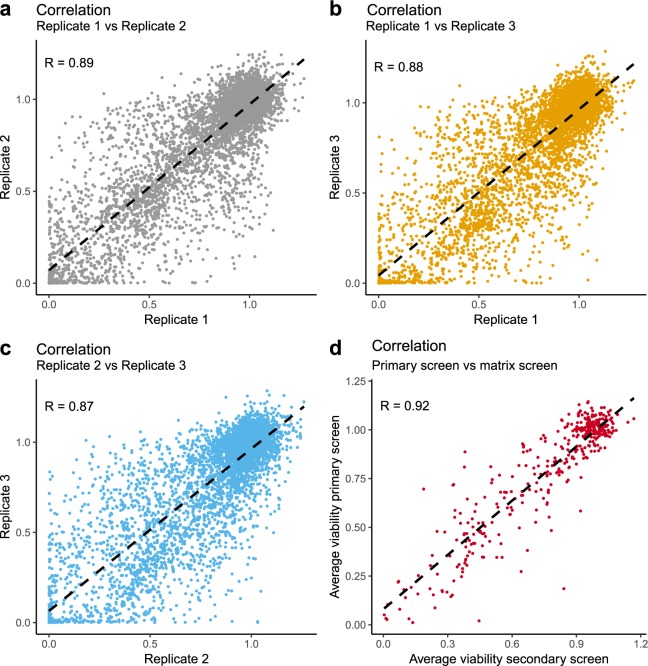
Scatter plots and computed Pearson correlation values for biological replicates. (**a**) 1 vs 2, (**b**) 1 vs 3 and (**c**) 2 vs 3. The correlation between shared conditions of primary and secondary screen are shown in (**d**).

To assess reproducibility between biological replicates, we calculated Pearson correlation coefficients for all measured data points (normalised). We observe a high correlation between all comparisons with a correlation of R = 0.89 between Replicate 1 vs Replicate 2, R = 0.88 between Replicate 1 vs Replicate 3 and R = 0.87 between Replicate 2 vs Replicate 3 of the primary screen (See Fig. 5a–c). In the viability data of the secondary screen we observed a correlation of R = 0.98 between Replicate 1 vs Replicate 2, R = 0.97 between Replicate 1 vs Replicate 3, and R = 0.98 between Replicate 2 vs Replicate 3. In the confluency data of the secondary screen we observed a correlation of R = 0.90 between Replicate 1 vs Replicate 2, R = 0.88 between Replicate 1 vs Replicate 3, and R = 0.95 between Replicate 2 vs Replicate 3. The average viability of shared data points between the primary and secondary screen showed a correlation of R = 0.92 (Fig. 5d). This variation is within the expected variation in high throughput cellular viability assays^11,26^.

### Synergistic combinations in primary screen

The primary screen led to the identification of 55 synergies according to the chosen cut-off from 1368 total combinations for all cell lines. An analysis of the list showed that 23 unique combinations were classified as synergistic across seven unique cell lines, with no synergistic combination in the MDA-MB-468 cell line. The inhibitors PI (targets PIK3CA/B/D, mTOR and DNA-PK) and SF (target PTEN) were identified to be involved in the majority of synergistic combination. The PI inhibitor was identified as synergistic with BI (target MAPK14) and with D1 (target RPS6KA1/2/3/6) in more than half of the tested cell lines. Further synergistic drug pairs sorted by occurrence across cell lines are G2 (target PDPK1), JN (targets MAPK8/9/10), P5 (target SYK) and AK (targets AKT1, AKT2, AKT3).

### Synergistic combinations in secondary screen

As the PI inhibitor was identified as one of the top drugs involved in synergistic combinations, it was chosen as the anchor drug for the secondary screen. We combined the PI inhibitor with the top four synergistic drug pairs judged by occurrence across cell lines. We also included PI in combination with PD (targets MAP2K1/2), to investigate synergistic behaviour of drug concentrations outside the concentration ratios tested in the primary screen. This was motivated by the fact that drugs targeting PI3K and MAP2K1/2 have been previously reported to be synergistic in several cell lines^18,19^. Additionally, we tested PI in combination with 5Z a combination previously identified as synergistic in the AGS cell line in a previous study by our group^15^ and Morris *et al*.^16^.

Results from the secondary screen confirmed combinations as being synergistic or antagonistic in most cell lines. We confirmed the synergistic effect of PI-G2 in SF-295, SW-620 and UACC-62 cells. Additionally, we observed a synergistic response in A498 and AGS cells in the secondary screen. The combination PI-BI was also redetected as synergistic in five of the cell lines, while for two cell lines the combination did not show a greater effect compared to single drug treatment in the secondary screen. For two combinations, that were not classified as synergistic in the primary screen, PI-5Z and PI-PD, we could observe synergistic responses in the secondary matrix design screen. The PI-5Z combination was synergistic in two cell lines including AGS cells, confirming previous findings^15,16^. The PI-PD combination (targets PIK3CA/B/D, mTOR and DNA-PK - MAP2K1/2) was found to be synergistic in five cell lines and non-synergistic in three cell lines including the two colorectal cancer cell lines SW-620 and COLO 205. While colorectal cancer cell lines have been frequently observed to display synergistic response to combined inhibition of MAP2K1/2 (MEK1/2) and PI3K^27–30^, weaker synergy has previously been observed in SW-620 and COLO 205 cells^28^.

## Usage Notes

Other users may investigate alternative methods for synergy quantification or another threshold using the provided data. To assist other researchers in visualising and analysing the dataset from the primary screen described herein we have prepared files that can be uploaded to the web-service CImbinator (http://rbbt.bsc.es/CImbinator/CombinationIndex) from which several synergy metrics can be computed and visualised^31^.

## Data Availability

The data was processed using R 3.5.2 and RStudio Version 1.1.463. The following packages were used for data processing “tidyr” (0.8.3), “dplyr” (0.8.1), “ggrepel” (0.8.1) and “stats” (3.5.2); and data visualization “RColorBrewer” (1.1-2), “gplots” (3.0.1.1), “ggplot2” (3.2.0), “ComplexHeatmap”^32^ (1.20.0), “ggpubr” (0.2) and “gridExtra” (2.3). All code applied for processing and visualising the data is provided at the figshare deposit^25^.

## Online-only Table

**Online-only Table 1:** List of inhibitors.

Abbreviation	Inhibitor	Primary target(s) (given by provider)	Term Accession Number	Term Source REF	PubChem CID	Formula	InChiKey	Provider
5Z	(5Z)-7-oxozeaenol (LL-Z1640-2)	MAP3K7, MAP2K1	CHEBI:67559	CHEBI	9863776	C19H22O7	NEQZWEXWOFPKOT-BYRRXHGESA-N	Enzo Diagnostics
60	BI605906 (BIX02514)	IKBKB			23652660	C17H22F2N4O3S2	IYHHRZBKXXKDDY-UHFFFAOYSA-N	MRC
AK	Akt Inhibitor VIII (AKTi-1,2)	AKT1, AKT2, AKT3			135398501	C34H29N7O	IWCQHVUQEFDRIW-UHFFFAOYSA-N	Merck Millipore
BI	Doramapimod (BIRB0796)	MAPK14, MAPK11	CHEBI:40953	CHEBI	156422	C31H37N5O3	MVCOAUNKQVWQHZ-UHFFFAOYSA-N	Axon Medchem
CT	CHIR 99021 (CT99021)	GSK3A, GSK3B	CHEBI:91091	CHEBI	9956119	C22H18Cl2N8	AQGNHMOJWBZFQQ-UHFFFAOYSA-N	Sigma-Aldrich
D1	BI-D1870	RPS6KA1, RPS6KA3, RPS6KA2, RPS6KA6			25023738	C19H23F2N5O2	DTEKTGDVSARYDS-UHFFFAOYSA-N	Selleckchem
D4	D4476	CSNK1A1, TGFBR1	CHEBI:91448	CHEBI	6419753	C23H18N4O3	DPDZHVCKYBCJHW-UHFFFAOYSA-N	Bio-Techne
F4	10058-F4	MYC			1271002	C12H11NOS2	SVXDHPADAXBMFB-JXMROGBWSA-N	Sigma-Aldrich
G2	GSK2334470	PDPK1	CHEBI:91465	CHEBI	46215815	C25H34N8O	QLPHOXTXAKOFMU-WBVHZDCISA-N	Sigma-Aldrich
G4	GSK-429286	ROCK1	CHEBI:91332	CHEBI	11373846	C21H16F4N4O2	OLIIUAHHAZEXEX-UHFFFAOYSA-N	Sigma-Aldrich
JN	JNK-IN-8 (JNK Inhibitor XVI)	MAPK8, MAPK9, MAPK10			57340686	C29H29N7O2	GJFCSAPFHAXMSF-UXBLZVDNSA-N	Merck Millipore
P5	PRT 062607 (P505-15)	SYK			44462758	C19H23N9O	TXGKRVFSSHPBAJ-JKSUJKDBSA-N	Axon Medchem
PD	PD0325901	MAP2K1, MAP2K2	CHEBI:88249	CHEBI	9826528	C16H14F3IN2O4	SUDAHWBOROXANE-SECBINFHSA-N	Sigma-Aldrich
PI	PI-103	PIK3CA, PIK3CB, PIK3CD, PIK3CG, MTOR, PRKDC	CHEBI:90524	CHEBI	9884685	C19H16N4O3	TUVCWJQQGGETHL-UHFFFAOYSA-N	Selleckchem
PK	Toxoflavin (PKF118–310)	CTNNB1	CHEBI:80729	CHEBI	66541	C7H7N5O2	SLGRAIAQIAUZAQ-UHFFFAOYSA-N	Sigma-Aldrich
RU	Ruxolitinib (INCB18424)	JAK1, JAK2	CHEBI:66919	CHEBI	25126798	C17H18N6	HFNKQEVNSGCOJV-OAHLLOKOSA-N	Selleckchem
SB	SB-505124	TGFBR1, ACVR1B, ACVR1C			56924523	C20H21N3O2 · xHCl · yH2O	DIDCCMVWCVRTNB-UHFFFAOYSA-N	Sigma-Aldrich
SF	SF1670	PTEN			9926586	C19H17NO3	VZQDDSYKVYARDW-UHFFFAOYSA-N	Sigma-Aldrich
ST	Stattic	STAT3	CHEBI:86989	CHEBI	2779853	C8H5NO4S	ZRRGOUHITGRLBA-UHFFFAOYSA-N	Sigma-Aldrich

Drugs are annotated by PubChem CID, InChiKey and CHEBI ID. Further drug target by provider, provider and formula are recorded. An extended table recording target information for each inhibitor is also available in the figshare deposit^25^.
